# *In-vivo* thermodynamic exploration of gas-based intraperitoneal hyperthermia

**DOI:** 10.3389/fonc.2022.925724

**Published:** 2022-08-29

**Authors:** Agata Diakun, Tanja Khosrawipour, Agata Mikolajczyk-Martinez, Piotr Kuropka, Jakub Nicpoń, Zdzisław Kiełbowicz, Przemysław Prządka, Bartłomiej Liszka, Shiri Li, Hien Lau, Wojciech Kielan, Veria Khosrawipour

**Affiliations:** ^1^ 2nd Department of General Surgery and Surgical Oncology, Wroclaw Medical University, Wroclaw, Poland; ^2^ Department of Surgery (A), University-Hospital Düsseldorf, Heinrich-Heine University, Düsseldorf, Germany; ^3^ Department of Biochemistry and Molecular Biology, Faculty of Veterinary Sciences, Wroclaw University of Environmental and Life Sciences, Wroclaw, Poland; ^4^ Department of Biostructure and Animal Physiology, Wroclaw University, Wroclaw University of Environmental and Life Sciences, Wroclaw, Poland; ^5^ Department of Surgery, Faculty of Veterinary Sciences, Wroclaw University of Environmental and Life Sciences, Wroclaw, Poland; ^6^ Division of Colon and Rectal Surgery, Department of Surgery, New York Presbyterian Hospital- Weill Cornell College of Medicine, New York, NY, United States; ^7^ Department of Surgery, University of California, Irvine, CA, United States; ^8^ Department of Surgery, Petrus-Hospital Wuppertal, Wuppertal, Germany

**Keywords:** Hyperthermia, thermodynamics, peritoneal metastases, intraperitoneal, chemotherapy, colorectal cancer

## Abstract

**Background:**

While hyperthermic intraperitoneal (i.p) applications are highly efficient in treating peritoneal metastases (PM), they are currently limited to temperatures of 41 – 43° Celsius (C). First data on gas-based i.p. hyperthermia is promising, as this novel method allows a significant temperature rise in superficial peritoneal layers without increasing core temperatures. Until now, key mechanisms of this novel tool, e.g. thermodynamic energy transfer, have not been investigated. This study aims to explore the volume of thermodynamic energy transfer during gas-based i.p. hyperthermia at 48-50°C and its peritoneal effects.

**Methods:**

For this study, three swine were subjected to gas-based i.p. hyperthermia at varying temperatures (48°, 49° and 50°C) in a diagnostic laparoscopy setting with a high-flow air stream. Temperatures of the i.p. cavity, in- and outflow airstream at the trocar were measured and the thermodynamic energy transfer was calculated. Tissue samples were collected on postoperative day 7 for histopathologic analyses.

**Results:**

According to our data, temperatures within the intraabdominal cavity and at the outflow site remain relatively stable at < 40°C. An increase in thermodynamic energy transfer is observed with increasing applied temperatures. Gas-based i.p. hyperthermia induced capillary coagulation and white blood cell infiltration within peritoneal layers.

**Conclusions:**

Gas-based i.p. hyperthermia is an innovative approach which enables the i.p. delivery of specific amounts of thermodynamic energy. Following this procedure, our data indicate remarkable histologic changes on the superficial peritoneal layer most likely attributable to the applied thermodynamic energy. Further studies are required to investigate how these findings can be applied in PM management.

## Introduction

Advanced peritoneal metastasis (PM) remains one of the key challenges in current surgical oncology. New concepts and approaches have been designed to improve the outcome of advanced, unresectable PM (1–5). With the introduction of Hyperthermic intraperitoneal chemotherapy (HIPEC) in combination with cytoreductive surgery (CRS) as a potentially curative treatment, hopes have been raised for some patients (6, 7). During HIPEC procedures, heated liquid chemotherapy is introduced into the abdominal cavity to eliminate remaining microscopic tumor cells after CRS (8). This effect is achieved by the combination of hyperthermia and chemotherapy. In the HIPEC setting, the medium perfusate temperature and core body temperatures usually remain at around 40°Celsius (C) (9). The increase of central body or total organ temperature is not desirable. A recent study by Goldenshluger et al. (10) demonstrated that increases in core body temperature served as a positive predictor for postoperative complications following HIPEC procedures. In fact, the relatively low temperature gradient between the applied HIPEC solution and core body temperature not only reduce the risk of overheating of single organs, but also the entire body. The beneficial effects of local hyperthermia in PM management have been well documented (11–13). While the sensitivity of cancer cells to increasing hyperthermia has been extensively demonstrated (14, 15), and hyperthermia has shown to increase the response rate of cancer cells to chemo- and radiotherapy (16–19), some studies suggest that the observed clinical effects of hyperthermia might be related to the occlusion of neo-vascular tumor structures (20–22). Currently effective hyperthermic intraperitoneal (i.p.) chemotherapy is limited to temperatures of 42 - 43°C. Yet, a previous study has demonstrated that far higher temperatures can be applied when using a gaseous medium as a mean for heat transportation (23). In contrast to water, air has a much lower heat capacity of around 0.718 kj/liter °C, when considering a density of 1.127 kg/m^3^ at 40°C and atmospheric pressure. Close-range objects retain their temperature for a longer time when surrounded by a medium with an over 5000-fold decreased heat capacity. This concept has also been demonstrated in a biological setting (23). As a result, superficial tissue layers can be exposed to higher temperatures while deeper tissues basically remain unaffected (23). The *in-vivo* feasibility of gas-based i.p. hyperthermia with temperatures extending beyond 43°C has been recently demonstrated and further studies in this field are currently conducted. Until now, there has been no data on how much thermodynamic energy is transferred during a gas-based i.p. hyperthermic procedure with temperatures extending beyond 43°C. By means of this study, we intend to explore the extent of thermodynamic energy transfer during gas-based i.p. hyperthermia at 48-50°C as well as its potential heating impact on peritoneal tissues. Furthermore, we aim to analyze the structural effects of gas-based i.p. hyperthermia on the peritoneal surface from a pathohistological perspective.

## Material and methods

### *In-vivo* swine model

Three 65-day-old swine (Polish white flod) received gas-based i.p. hyperthermia at 48°, 49° and 50°C, respectively, in a diagnostic laparoscopy setting under a high-flow air stream of 15 liters per minute (l/min). The swine were part of a multicenter and multinational research study on peritoneal hyperthermia and dehydration. All animals received humane care in compliance with the Guide for the Care and Use of Laboratory Animals as published by the National Institutes of Health.

### Gas-based i.p. hyperthermia in the laparoscopic setting

**Figure 1:**
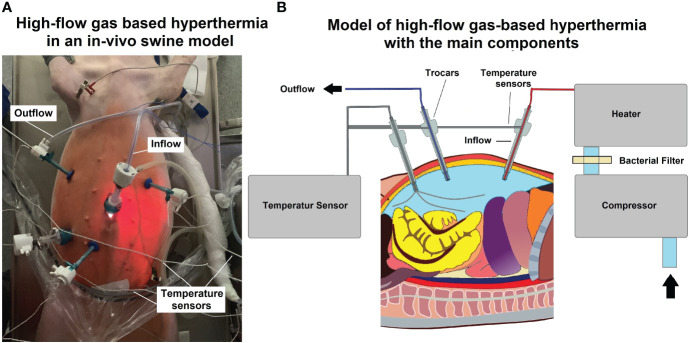
**(A)** Laparoscopy in the *in vivo* swine during high-flow gas-based hyperthermia. Multiple placements of temperature-sensors for intraoperative monitoring **(B)** Technical model of high-flow gas-based hyperthermia.

For the procedure, swine were placed under general anesthesia. Premedication was conducted using an intramuscular (i.m) injection of midazolam (0.3 mg/kg, WZF Polfa S.A., Poland), medetomidine (0.02 mg/kg, Cepetor 1 mg/ml, CP-Pharma Handelsgesellschaft, Germany) and ketamine (9 mg/kg, Ketamina 100 mg/ml, Biowet Puławy sp. z o.o., Poland) mixture. Analgesia was performed with Propofol at 1mg/kg. Swine were intubated and anesthesia was continued with isoflurane 1%. Additional analgesia was provided with fentanyl 2µg/kg and crystalloid fluid at 0.2-0.3 µg/kg/min. For the surgical procedure, swine were placed in a supine position. An infra-umbilical mini-laparotomy was performed and another one at about 8 cm distance to the first one. A 10 mm trocar (Kii^®^Balloon Blunt Tip System, Applied Medical, Rancho Santa Margarita, CA, USA) was inserted *via* the infra-umbilical trocar while several 5 mm trocars were placed at the other sites after insufflation (**Figure 1A**). The abdominal cavity was insufflated with filtered room air using a tube entering the central 10 mm trocar. An initial diagnostic check-up was conducted *via* laparoscopic imaging using a 5 mm camera system (Karl Storz 5mm/30° Laparoscope/Tuttlingen, Germany) and a 5 mm trocar. After visual confirmation and placement of multiple temperature sensors (**Figure 1B**), the high-flow air stream was turned on at 15 l/min for a total of 45 minutes. Intracavitary temperatures were monitored to detect the onset of critical intraabdominal heating. A total of four temperature sensors were placed in the abdominal cavity. One was placed in the lower quadrant, one in the right upper and one in the left upper quadrant, an additional probe was placed at close contact to the small intestine in the right central area but still within the cavity. In- and outflow temperatures were measured at the trocar site. Postoperatively, swine were monitored for seven days, and blood samples were drawn to detect possible postoperative complications.

### Analyzing the operative model for laparoscopic dehydration of the abdominal cavity

Based on mean temperatures at the in- and outflow site, we calculated the temperature difference. With a given flow rate of 15 l/min, the thermodynamic energy transfer was calculated based on the first law of thermodynamics (conversation of energy) adapted for the thermodynamic process. The calculation was based on the specific heat capacity of air at 1 bar (atmospheric pressure), which ranges between 0.718 - 0.7206 KiloJoule (kJ) per Kilogram (kg) air at 27 - 67° C, and air density at atmospheric pressure (1 bar) given with 1.271 Kg per m^3^ at 40° C. Based on the model of laparoscopic surface area quantification by Khosrawipour et al. (24), we used 16 dm^2^ as the interacting peritoneal surface to estimate the extent of heat buildup on the peritoneal layer.

### Euthanization

Seven days after the procedure, the swine were subjected to euthanization and autopsy. For this purpose, swine were premedicated with an i.m. injection of midazolam (0.1 mg/kg, Midanium 5 mg/ml, WZF Polfa S.A., Poland), medetomidine (0.02 mg/kg, Cepetor 1 mg/ml, CP-Pharma Handelsgesellschaft, Germany) and ketamine (8 mg/kg, Ketamina 100 mg/ml, Biowet Puławy sp. z o.o., Poland) mixture. After that, swine were euthanized with an intravenous injection by Sodium Pentobarbital with Pentobarbital (50mg/kg with 12 mg/kg, Morbital 133.3 mg/ml + 26.7 mg/ml, Biowet Pulawy Sp. z o.o., Poland) according to current recommendations (18). Post-mortem swine cadavers were placed in supine position and a median laparotomy was performed. Peritoneal tissue samples were removed from distinct locations within the abdomen for further histological analyzes. Additionally, hepatic samples were collected.

### Histopathological examination of peritoneal tissue and liver samples

Samples were fixed for 48 hours in 10% neutral buffered formalin (cat# 22-026-354, Fisher Scientific). Formalin-fixed samples were prepared for paraffin processing by serial dehydration in increasing concentrations of ethanol solutions using a tissue processor (Leica TP1020, Leica Microsystems). After preparation, tissues were embedded in paraffin wax using a tissue embedder (Leica EG 1150C, Leica Microsystems). Paraffin-embedded tissue blocks were sectioned into 5µm sections on a microtome (Leica RM 2255, Leica Microsystems). 5µm sections were stained with HE. All slides were imaged using an inverted microscope (Nikon Ti-E Widefield microscope, Nikon Instruments Inc.).

### Graphic design

For the graphics provided, multiple graphic programs were used. These programs included Inkscape 1.0.1,2020, GNU, USA as well as programs provided by Windows office 2019, Microsoft.

### Statistical analysis

Three swine were exposed to mean temperature levels of 48°C, 49°C and 50°C.

Data are presented as the mean and the standard deviation unless otherwise indicated. A boxplot was used to visualize heat distribution. Mean heat-values were used to effectively calculate the thermodynamic gradient and energy transfer. Liver-samples for each swine were removed at 3 different, exposed sites within the laparoscopic cavity. Further peritoneal samples were taken at a total of 8 different sites per swine.

The student t-test was used to compare independent groups. Probability (p) values were considered as follows: *=p<0.01, and #=p>0.05, with p-value <0.05 considered to be statistically significant.

## Results

### Thermodynamics of gas-based i.p. hyperthermia

**Figure 2:**
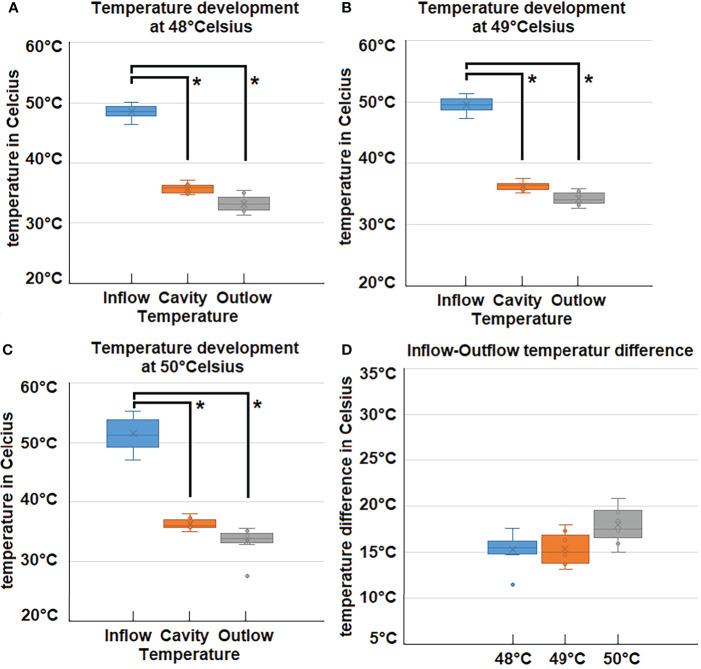
**(A–C)** Measured temperatures during laparoscopy at 48°C, 49°C and 50°C inflow, cavity- and outflow temperatures. **(D)** Difference (Delta) of Inflow and Outlfow temperatures for high-flow hyperthermia at 48°C, 49°C and 50°C. *p < 0.05.

**Figure 3:**
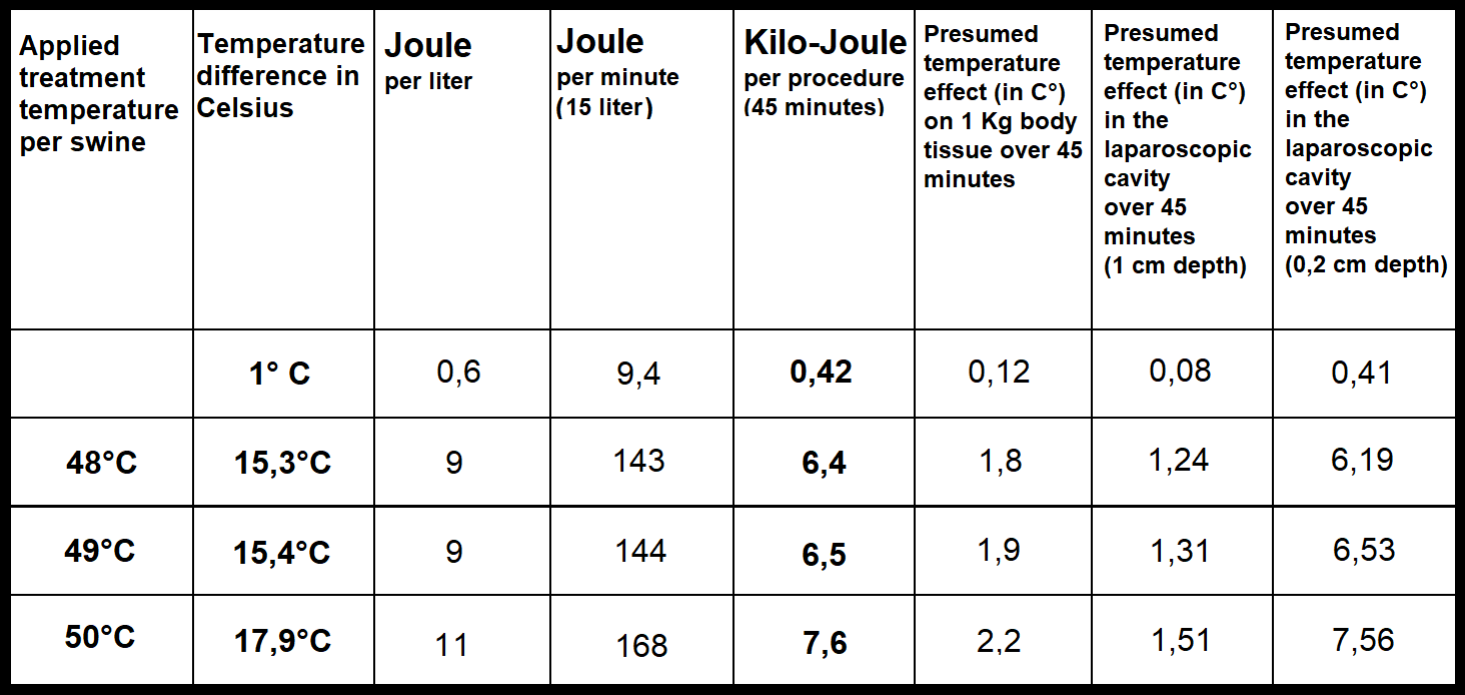
Calculated energy transfer during high-flow gas-based hyperthermia at 48°C, 49°C and 50°C based on delta (inflow and outflow temperature difference) and flow-rate).

The experimental protocol was successfully executed. No intra- or postoperative complications were observed. All swine were extubated without problems. No distress or signs of abnormal behavior were detected. Intraoperative temperature measurements during the 45-minute procedure revealed that inflow, outflow and cavity temperature remained constant (**Figures 2A–C**). While the inflow temperature varies around the targeted 48°C, 49°C, and 50°C, the outflow temperature remained constant in each case within a narrow margin of 33.2 ± .1.3°C (48°C), 33.4± 1.9 C° (49°C) and 33.6 ± 2°C (50°C). The temperature difference (inflow vs. outflow) therefore increases with higher inflow temperature (**Figure 2D**). The mean difference is 15.3°C (48°C), 15.4°C (49°C) and 17.9°C (50°C) (**Figure 3**). For the presented 45-minute procedure, the thermodynamic energy transfer was calculated to be 6.4 kJ for the 48°C, 6.5 kJ for 49°C and 7.6 kJ for 50°C application, respectively (**Figure 3**). The mean thermodynamic energy transfer was approximately 0.42 kJ/°C during the 45-minute application. Based on these calculations, the presumed temperature effects on tissue were estimated. The temperature effect of 7.6 kJ, which corresponds to the energy transfer during a 45-minute gas-based hyperthermia procedure at 50°C on a 1 kg tissue sample, corresponds to 2.2°C (**Figure 3**). Assuming the effect of the thermodynamic energy of 7.6 kJ would have been limited on the most superficial 0.2 cm vs. 1 cm of the peritoneal surface (16 dm^2^), the estimated temperature effect would have been 1.51°C vs. 7.56°C (**Figure 3**).

### Histopathological examination, liver tissue and serum parameters

**Figure 4:**
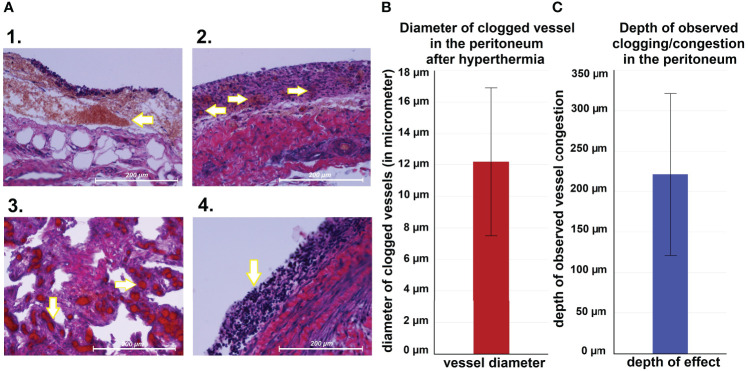
**(A)** Microscopic peritoneal changes following high-flow gas-based hyperthermia. 1. small „hyperthermic” petechia, 2. Micropetechia on the peritoneal surface. 3. Clotting of capillary vessels on the peritoneal surface. 4. Peritoneal white blood cell infiltration. **(B)** Measured diameter of clotted vessels on the peritoneal surface. **(C)** Depth land extent of vessel clotting.

**Figure 5:**
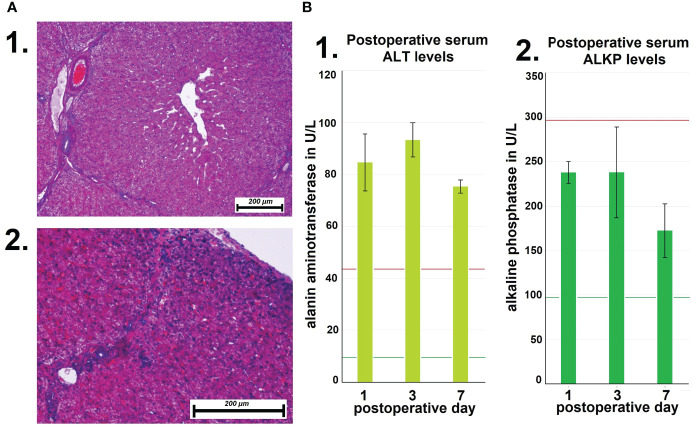
**(A)** Microscopic changes of liver tissue following high-flow gas-based hyperthermia. 1. Deep tissue samples from the liver without pathologies. 2. Vascular clotting in capillaries close to the liver capsule. **(B)** Mean postoperative serum levels of alanin aminotransferase (ALT) at day 1, 3 and 7. Mean postoperative serum levels of alkaline phosphatase (ALT).

Following autopsy, swine tissue samples were removed from multiple locations within the peritoneum. Areas exposed to the hyperthermic laparoscopic space were compared to unexposed peritoneal samples of the same swine. Specific changes in exposed areas were analyzed and documented (**Figure 4A**). Hematoxylin and eosin (HE) staining depicted changes 7 days after i.p. hyperthermia. Spots of micropetechial bleeding were detected within the superficial peritoneal layer (**Figure 4A1**). Additionally, within the first few hundred millimeter of the peritoneal surface, the capillary system (**Figures 4B, C**) showed signs of occlusion by clotting (**Figures 4A1, 2**). In the peritoneal tissue exposed to the laparoscopic cavity, peritoneal edema, and an increase in white blood cell infiltration were detected (**Figure 4A**). In only a few cases, damage to muscle membrane cells of the intestine were observed. Unexposed peritoneal tissue samples did not show any specific changes, nor did they present signs of edema or white blood cell infiltration. The phenomenon of capillary clotting was also observed in liver tissues a few hundred millimeter close to the liver capsule (**Figure 5A1 vs A2**). With respect to serum related liver parameters alanine aminotransferase (ALT) and alkaline phosphatase (ALKP), only an ALT increase was noticed on postoperative days 1, 3 and 7. The peak ALT elevation was on day 3 (**Figures 5B1, B2**).

## Discussion

Continuous efforts are made to improve the management and therapeutic options for PM patients. These efforts include attempts to apply new physical principles to the already existing pharmacological options to enhance local antitumoral effects (25–29). Some of these concepts include irradiation (30–32), high-intensity ultrasound (33–35), nanoparticles (36) as well as the application of new substances (37, 38). Hyperthermia notably impacts PM management as it demonstrates great efficacy in enhancing antitumoral effects when combined with chemotherapy or radiation, without causing disproportionate additional side effects (39–41). Until now, hyperthermia is usually applied using water-based fluid solutions, which display their own set of limitations due to the unique physical properties of water. However, based on the different physical properties of air, new therapeutic constellations might be available. The presented data indicates that the abdominal cavity can be exposed to gas-based hyperthermia exceeding temperatures of 43°C with a defined thermodynamic energy dose. Although we do not observe any relevant general temperature increase within the peritoneal cavity or systemically in the treated patient, we do observe the effect of the applied temperature dose on the upper superficial peritoneal layer.

There are a variety of alternative explanations for this phenomenon. Of course, the most obvious explanation is that the measured intraperitoneal temperatures are as presented and the observed changes on the peritoneum are merely related to dehydration effects of the peritoneum. Another possibility is that the temperature effect is limited to the first few hundred millimeters of the peritoneal layer, thus only affecting the superficial peritoneal layer. In this scenario, the thermodynamic energy may be insufficient to heat up and cause a measurable increase in the heat sensor due to its own heat-capacity. Another explanation is that low air velocity around the intracavitary temperature probes and low thermodynamic air conductivity does not heat up the temperature probes. The temperature sensors at the high-velocity sites at the outflow and inflow are affected in the same way. The last possible explanation is that the thermodynamic energy applied on the peritoneal surface is wasted in chemical processes which change the peritoneal tissue in the area. When we look at alternative gas-mediums and their relationship with the applied regular air in our model, we can estimate the changes in energy transfer assuming the same inflow-outflow temperatures as set values. Based on the available data on the isobaric volumetric heat capacity of the listed gases compared to room air, the heat carrying capacity of the medium at the same volume would increase or decrease approximately in the following manner: CO_2_: +28%; N_2_: -2%; O_2_: +7%, Helium: -24%. To determine to what extent the flow rate influences the temperature difference between the outflow and inflow, further separate studies are required. However, as surface velocity is a factor in the heat transfer from one medium to another, we must assume that this will be a relevant factor. In fact, the contribution of dehydration to the observed effects should be separately analyzed and studied. This requires a separate study on normothermic dehydration on a high-flow laparoscopic model. We believe that with this presented study, we have a strong argument to plan and argue for future and more comprehensive *in-vivo* studies.

However, the extent to which this novel tool is beneficial and or relevant for PM treatment must be further studied. In fact, capillary occlusion of neovascular structures could be a highly relevant mechanism to halt invasive cell growth.

Yet, it has been recognized that there is still no experience and only little understanding of the effects of a hyperthermic capnoperitoneum due to the physical challenges created by air as a carrier medium with an extremely low-heat capacity and unique physical qualities. More basic research must be conducted to improve the understanding and management of gas-based hyperthermia. Further studies are required to investigate whether gas-based i.p. hyperthermia can serve as an independent therapeutic option for PM treatment or if its use as an add-on therapy is more favorable (41–44). Gas-based i.p. hyperthermia with temperatures extending beyond 43°C is feasible and might serve as a new therapeutic option for advanced PM. This novel tool has the potential to create a temperature gradient along the peritoneal surface to apply hyperthermia, stop neo-vascularization and further dehydrate metastatic nodules along the peritoneum (24) to decelerate overall disease progression. However, applicational, biological and technical aspects of this novel approach must be further analyzed.

## Data availability statement

The original contributions presented in the study are included in the article/supplementary material. Further inquiries can be directed to the corresponding authors.

## Ethics statement

The experiments were approved (Nr 030/2021/P2) by the local Ethics Committee of Wroclaw University of Environmental and Life Sciences, Wroclaw, Poland as well as the local Board on Animal Welfare.

## Author contributions

AD: Study design, laboratory analysis, data acquisition, manuscript drafting. TK: manuscript drafting and critical revision for important intellectual content of the manuscript. AM-M: Study design, laboratory analysis, data acquisition. PK: laboratory analysis, data analyses. JN: Study design, drafting and critical revision for important intellectual content of the manuscript. ZK: laboratory analysis, data acquisition. PP: Study design, laboratory analysis, data acquisition. BL: Study design, laboratory analysis, data acquisition. SL: data acquisition and manuscript drafting. HL: data analyses and intellectual content of the manuscript. WK: manuscript drafting and critical revision for important intellectual content of the manuscript. VK: Supervision on study design, laboratory analyses, conception of the study and manuscript drafting. All authors contributed to the article and approved the submitted version.

## Funding

This study was funded by institutional funds from the Departments of Biochemistry and Molecular Biology, Veterinary surgery as well as by the Department of surgery (A), university hospital of Düsseldorf.

## Acknowledgments

We thank Dr. Thomas Siegert from the Department of Extraterrestrial physics at the Max-Planck Institute, Garching, near Munich Germany for his counseling on thermodynamics.

## Conflict of interest

The authors declare that the research was conducted in the absence of any commercial or financial relationships that could be construed as a potential conflict of interest.

## Publisher’s note

All claims expressed in this article are solely those of the authors and do not necessarily represent those of their affiliated organizations, or those of the publisher, the editors and the reviewers. Any product that may be evaluated in this article, or claim that may be made by its manufacturer, is not guaranteed or endorsed by the publisher.
